# B-RAF Mutant Alleles Associated with Langerhans Cell Histiocytosis, a Granulomatous Pediatric Disease

**DOI:** 10.1371/journal.pone.0033891

**Published:** 2012-04-10

**Authors:** Takeshi Satoh, Alexander Smith, Aurelien Sarde, Hui-chun Lu, Sophie Mian, Celine Trouillet, Ghulam Mufti, Jean-Francois Emile, Franca Fraternali, Jean Donadieu, Frederic Geissmann

**Affiliations:** 1 School of Medicine, Centre for Molecular and Cellular Biology of Inflammation (CMCBI), King's College London, London, United Kingdom; 2 Haematology Department, King's College London, London, United Kingdom; 3 Randall Division of Molecular Biophysics, King's College London, London, United Kingdom; 4 Hopital Ambroise-Pare, Pathology department, AP-HP, Paris, France; 5 Centre de Référence de l'Histiocytose, Hopital d'Enfants Armand Trousseau, Pediatric Hematology Unit, AP-HP, Paris, France; Kaohsiung Chang Gung Memorial Hospital, Taiwan

## Abstract

**Background:**

Langerhans cell histiocytosis (LCH) features inflammatory granuloma characterised by the presence of CD1a+ dendritic cells or ‘LCH cells’. Badalian-Very et al. recently reported the presence of a canonical ^V600E^B-RAF mutation in 57% of paraffin-embedded biopsies from LCH granuloma. Here we confirm their findings and report the identification of two novel B-RAF mutations detected in LCH patients.

**Methods and Results:**

Mutations of B-RAF were observed in granuloma samples from 11 out of 16 patients using ‘next generation’ pyrosequencing. In 9 cases the mutation identified was ^V600E^B-RAF. In 2 cases novel polymorphisms were identified. A somatic ^600DLAT^B-RAF insertion mimicked the structural and functional consequences of the ^V600E^B-RAF mutant. It destabilized the inactive conformation of the B-RAF kinase and resulted in increased ERK activation in 293 T cells. The ^600DLAT^B-RAF and ^V600E^B-RAF mutations were found enriched in DNA and mRNA from the CD1a+ fraction of granuloma. They were absent from the blood and monocytes of 58 LCH patients, with a lower threshold of sequencing sensitivity of 1%–2% relative mutation abundance. A novel germ line ^T599A^B-RAF mutant allele was detected in one patient, at a relative mutation abundance close to 50% in the LCH granuloma, blood monocytes and lymphocytes. However, ^T599A^B-RAF did not destabilize the inactive conformation of the B-RAF kinase, and did not induce increased ERK phosphorylation or C-RAF transactivation.

**Conclusions:**

Our data confirmed presence of the ^V600E^B-RAF mutation in LCH granuloma of some patients, and identify two novel B-RAF mutations. They indicate that ^V600E^B-RAF and ^600DLAT^B-RAF mutations are somatic mutants enriched in LCH CD1a^+^ cells and absent from the patient blood. Further studies are needed to assess the functional consequences of the germ-line ^T599A^B-RAF allele.

## Introduction

Langerhans cell histiocytosis (LCH) is a pediatric granulomatous disease with an incidence of four to eight cases per million children [1], [2], [3]. The clinical spectrum of LCH is remarkably broad, ranging from isolated skin or bone lesions to a disseminated disease that may require aggressive chemotherapy [1], [4], [5]. LCH can lead to severe dental and periodontal lesions [6]. LCH also frequently leads to diabetes insipidus [1], [2], [3]. Apart from diabetes insipidus, central nervous system involvement in Langerhans cell histiocytosis (LCH) is rare but represent a serious complication with neurological deterioration, including progressive cerebellar ataxia [7].

LCH lesions feature granulomatous collections of immature CD1a+ langerin/CD207+ DC (‘LCH cells’) presumed to be pathologic [8], [9], [10], admixed with abundant eosinophils [11], polyclonal T cells including abundant FoxP3^+^ CD4^+^ T cells [12], activated macrophages and osteoclast-like multinucleated giant cells [13]. These granuloma are therefore heterogeneous in cellular composition as well as anatomical distribution. The pathophysiology of LCH is largely unknown [10], [14], although a genetic component is suggested by a higher concordance rate between monozygotic twins compared with dizygotic twins [15]. The tropism of skin lesions to flexures also suggests that external stimuli may trigger inflammation [16]. However, the nature of the initiating event(s), and the mechanisms of local tissue destruction by LCH and other inflammatory cells are still largely unknown.

Clonality of LCH granulomas has been reported in 1994 [17], [18]. Its significance remained controversial, since specific genetic abnormalities were not consistently observed [19], until recently. Progress came from the identification by Badalian-Very et al., of a ^V600E^B-RAF mutation by pyrosequencing of formalin-fixed, paraffin-embedded material, from 35 out of 61 archived specimens (57%) [20]. This ^V600E^B-RAF mutation is likely to be somatic, because a germ-line activating ^V600E^B-RAF allele is embryonic lethal in mice [21]. B-RAF is a protein kinase activated by ras-coupled receptor tyrosine kinases (RTK) that is central to signaling via the Mitogen Activated Kinase (MAPK) and phosphorylates its downstream target MEK and ERK kinases [22]. The RAS-RAF-MAPK pathway coordinates a large variety of cellular responses involved in development, cell cycle regulation, cell proliferation and differentiation, cell survival and apoptosis, and many other physiological processes, by transmitting extracellular signals to various nuclear, cytoplasmic and membrane-bound targets [22].

Data obtained from murine model using Cre-mediated activation of a conditionnal B-RAF allele indicate that ^V600E^B-RAF can contribute to tumour initiation [23]. For example, ^V600E^B-RAF induces high levels of cyclin D1-mediated cell proliferation. However, ^V600E^B-RAF also induces oncogene-induced senescence (OIS) that may restrain further development of the tumour [23], [24]. In human, somatic ^V600E^B-RAF mutation have been found in a number of benign and malignant tumors including non-malignant naevi [25], melanoma [26], colon and thyroid tumors [21], [22], [24], [26]. Thus ^V600E^B-RAF may represent a first step toward the development of a malignant tumor, although the presence of a ^V600E^B-RAF mutation is not synonymous with cancer.

Of note, the clinical features of LCH are not typical of cancer [10], [14] and LCH lesions frequently regress, either spontaneously or after local treatment [1], [4], [5]. In addition, LCH CD1a+ cells, which are presumed to be pathologic, very slowly proliferate in most patients [12], while the expansion of a monocyte or dendritic cell compartment, which represent candidate precursors for these CD1a+ cells of the granuloma, was not consistently observed in the blood of LCH patients [12], [27].

Identification of dysregulation of the RAS-RAF-MAPK pathway in LCH is nevertheless an important step towards a molecular understanding of the pathophysiology of this pediatric granuloma. It suggests possible novel therapeutic approaches, e.g. the use of B-RAF or MEK inhibitors. Moreover, if ^V600E^B-RAF was detectable in the patient's blood, this could be of use for diagnostic, monitoring of treatment efficacy, and potentially prognostic purposes.

We therefore sought firstly to confirm the finding of Badalian-Very using another methodology i.e. analyzing flow sorted CD1a+ cells from fresh LCH granuloma tissue instead of paraffin embedded biopsies. We then aimed to compare relative mutation abundance of V600E B-RAF mutations between CD1a+ and CD1a− cells from LCH granuloma, and whether mutations can be detected in the peripheral blood of patients. Finally we investigated whether additional B-RAF mutations can be found in LCH patients, to further reinforce the link between LCH and the RAS-RAF pathway.

## Results

### B-RAF polymorphisms associated with LCH

**Figure 1:**
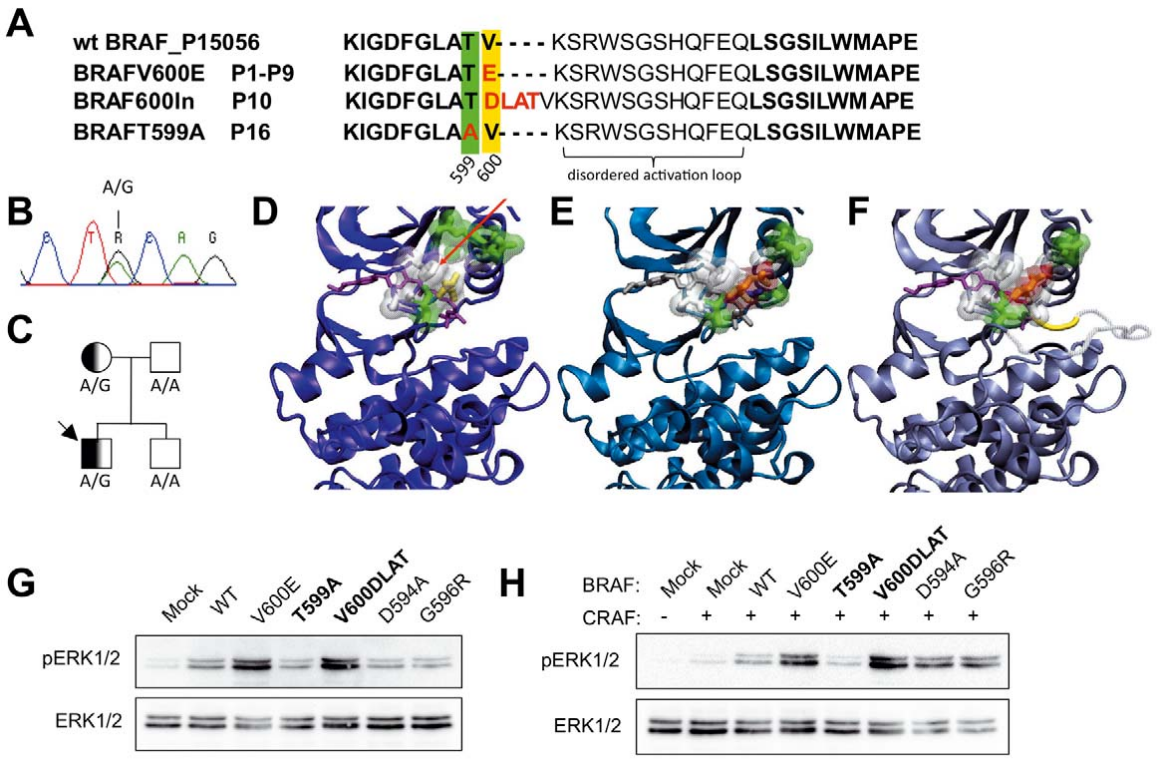
Analysis of B-RAF mutant. **A.** Sequence alignment, results from 454 pyrosequencing of granuloma cells from patients 1–10 and 16. **B.** ‘Sanger’ sequencing of patient 16 blood; A/G transition at nucleotide 1795. **C.** Pedigree of patient 16. Both the patient and his mother carry a ^T599A^B-RAF allele, while his father is ^wt^B-RAF. **D–E.** Comparison between ^wt^B-RAF 5P_15056 (D, purple), ^V600E^B-RAF structure (E, cyan) and the modeled mutant ^600DLAT^B-RAF (F, grey). In D, Val600 (yellow) forms a hydrophobic contact with Phe468 (red arrow). In E and F charged residues Asp and Glu (in orange) disrupt the hydrophobic network of interactions, stabilising the active conformation of the P-loop. In F, insertion Asp-Leu-Ala-Thr shifted Val600 and disrupt the hydrophobic cluster. **G, H.** MEK phosphorylation in 293 T cells. 293 T cells were transiently transfected with with mock or B-RAF mutant expressing vectors (WT, V600E, T599A, 600DLAT, D594A, G596R), and with (H) or without (G) wtC-RAF. Twenty-four hours after transfection, the medium was changed to serum-free DMEM, followed by further 18 hours culture. Total cell lysates were immunoblotted with the indicated antibodies.

We investigated the presence of B-RAF mutations by ‘next generation’ pyrosequencing (Roche GS FLX 454) in a series of 16 granuloma samples obtained at diagnosis from 16 patients with LCH from a cohort followed in the French LCH Registry (Tables 1 & 2). Among granuloma samples 11/16 carried B-RAF mutations as detected by pyrosequencing of granuloma genomic DNA (gDNA) and cDNA (Table 1). In 9/11 cases (patients #1–9) we found a g1799 T>A transition resulting in the previously described ^V600E^B-RAF mutation [20] (Figure 1 A). In one patient (patient #10) we found a novel in-frame insertion of 12 nucleotides, leading to the insertion of 4 amino acids (Asp-Leu-Ala-Thr, or DLAT) (Figure 1 A). In one other patient we observed an A>G transversion, producing a ^T599A^B-RAF allele (Table 2, Figure 1 A). The ^T599A^B-RAF and ^600DLAT^B-RAF alleles were not previously reported in the literature, dbSNP (v131), or in data from 1000 genomes project (Nov 10 release).

**Table 1:** Age, sex, and clinical characteristics of patients 1 to 16.

Patient#	NUP	age at diagnosis (y)	sex	clinical features
#1	1506614	7.9	F	bone
#2	1506751	9	M	bone
#3	2106401	0.26	M	skin, lung, lympn node
#4	2106143	0.5	M	LETTERER SIWE hematological dysfunction, bone, liver, spleen
#5	1406259	8.4	F	bone
#6	1506706	19	M	Skin
#7	2106169	0.438	F	LETTERER SIWE hematological dysfunction, bone, liver, spleen, pituitary
#8	1406253	8.6	F	bone
#9	1406254	7.6	M	bone
#10	1406015	0	F	LETTERER SIWE hematological dysfunction, bone, liver, spleen, pituitary, lung, lymph nodes
#11	1406210	10	M	bone
#12	1506008	0.97	F	LETTERER SIWE hematological dysfunction, bone, lung, liver, spleen
#13	1406215	5.3	M	bone
#14	1406247	7.59	F	bone, lymph node
#15	1506766	11.79	F	bone
#16	1506646	1	M	skin, bone

**Table 2:** Presence and relative abundance of B-RAF mutant clones identified in granuloma and blood from patients with LCH.

		granuloma							
		total	gDNA		cDNA		blood, gDNA		
Patient#	NUP	gDNA	CD1a+	effluent	CD1a+	effluent	whole blood	CD14+	CD14−
#1	1506614	V600E 13,5%					wt	wt	wt
#2	1506751	V600E 29,4%					wt		
#3	2106401	V600E 16%							
#4	2106143		V600E 35%		V600E 40%				
#5	1406259		V600E 38%	V600E 22%	V600E 46%	V600E 24%			
#6	1506706	V600E 8%							
#7	2106169	V600E 9%							
#8	1406253		V600E 21%	V600E 8%	V600E 48%	V600E 6%			
#9	1406254		V600E 35%	V600E 23%	V600E 47%	V600E 35%			
#10	1406015		600DLAT 11%	600DLAT 5%	600DLAT 28%	600DLAT 11%			
#11	1406210	wt							
#12	1506008	wt							
#13	1406215	wt							
#14	1406247	wt							
#15	1506766	wt						wt	wt
#16	1506646	T599A 44%					T599A 37%	T599A 37%	T599A 45%

### Somatic B-RAF mutations

The ^V600E^B-RAF and ^600DLAT^B-RAF, mutations were detected in granuloma from patients 1–10 with a high relative mutation abundance (RMA) (Table 2) We therefore investigated whether these alleles were germ-line or somatic mutants, and which cellular fraction of the granuloma was bearing the mutation. In 4 cases of ^V600E^B-RAF mutation (Patients 4, 5, 8, 9) and in the sample carrying the ^600DLAT^B-RAF mutation (patient #10) (Table 2), CD1a+ cells were enriched from fresh granuloma tissue using antibody-coupled beads [8]. The CD1a-depleted fractions (Effluent, Table 2) were also collected in 4 cases (Patients 5, 8, 9, 10, Table 2). Genomic DNA and cDNA were extracted from all samples and analysed by next generation sequencing. In all cases B-RAF mutated alleles were enriched in the CD1a+ fraction at the genomic DNA and cDNA level, in comparison with the CD1a-depleted fractions, with a relative mutation abundance of up to 48% (Table 2), suggesting that the CD1a+ ‘LCH cells’ carry a heterozygous B-RAF mutant allele, though we did not assess heterozygosity at the single cell level. We then investigated whether ^V600E^B-RAF or ^600DLAT^B-RAF mutations could be found in the patients circulating myeloid or lymphoid cells, either because of a germ-line mutation, or because of a mosaicism in bone marrow progenitors. Analysis by pyrosequencing of whole blood and of purified monocytes (CD14+) and lymphocytes (CD14−) from patient 1 indicated the absence of detectable B-RAF mutations, with a lower threshold of sequencing sensitivity of 1%–2% relative mutation abundance. Analysis of whole blood from patient 2 with the same method also failed to detect a ^V600E^B-RAF mutation. Blood samples were not available for the 8 other patients with a ^V600E^B-RAF or ^600DLAT^B-RAF mutation.

We therefore investigated the presence of B-RAF mutations in peripheral blood mononuclear cells of an additional series of LCH patients for whom blood samples were available either at diagnosis or relapse (n=22) (Table 3), or under treatment (n=32). Neither ^V600E^B-RAF or ^600DLAT^B-RAF were detectable by pyrosequencing in these 56 peripheral blood mononuclear cell samples (Tables 2 & 3, and data not shown). Unfortunately granuloma samples were not availables for these patients, however based on the frequency of B-RAF mutation in LCH granuloma, 57% in the Badalian-Very study, and 11/16 (68%) in the present study, it is likely that several of the 56 patients had a B-RAF mutation in their granuloma.

**Table 3:** Age, sex, clinical features, and molecular findings in 22 patients with available blood samples at the time of diagnosis or relapse.

patient #	NUP	Clinical features	status at the time of blood sample	BRAF		
				whole blood	CD14+	CD14−
#17	1406220	bone skin, ENT, pituitary, neuro	active/progressive disease	na	wt	wt
#18	1506752	skin	active/progressive disease	wt	na	na
#19	1506604	Bone, skin, hematological dysfunction	active/progressive disease	wt	na	na
#20	1506648	Bone, skin, ENT, lung, liver, hematological dysfunction	active/progressive disease	wt	na	na
#21	1506882	Skin, ENT	active/progressive disease	wt	na	na
#22	1506863	Bone, skin, ENT, lung, liver, hematological dysfunction	active/progressive disease	wt	na	na
#23	1506009	Bone, skin, ENT, nodes, pituitary, lung, liver, spleen, hematological dysfunction	active/progressive disease	wt	na	na
#24	1507109	skin, ENT, pituitary	active/progressive disease	wt	na	na
#25	2106005	Bone, skin, ENT, CNS mass lesion	active/progressive disease	wt	na	na
#26	1509003	bone	at diagnosis, before treatment	wt	na	na
#27	1506819	bone	at diagnosis, before treatment	wt	na	na
#28	1507093	bone	at diagnosis, before treatment	wt	wt	na
#29	1506957	lung	at diagnosis, before treatment	wt	na	na
#30	1506973	bone, pituitary	at diagnosis, before treatment	wt	na	na
#31	1507062	Skin	at diagnosis, before treatment	wt	wt	wt
#32	1506869	bone	1 MONTH DIAGNOSIS	wt	na	na
#33	1507096	bone	<2 MONTH DIAGNOSIS	na	wt	wt
#34	1506754	skin	2 MONTHS DIAGNOSIS	wt	na	na
#35	1506865	bone	2 MONTH DIAGNOSIS	wt	na	na
#36	1507084	skin	2 MONTH DIAGNOSIS	wt	wt	wt
#37	1506932	bone	3 MONTH DIAGNOSIS	wt	wt	na
#38	1506984	skin	3 MONTH DIAGNOSIS	wt	na	na

In aggregate, these data indicate that ^V600E^B-RAF and ^600DLAT^B-RAF mutations are detectable in granuloma but not in the blood, and thus strongly suggest they are somatic events. In addition, lack of detection of B-RAF mutations in the blood from patients 1 and 2 and from the 22 others patients for whom blood samples were available at diagnosis or relapse suggests that detection of potential circulating B-RAF mutated cells will require a threshold of sensitivity below 1%.

### Germline B-RAF polymorphisms

In contrast, the ^T599A^B-RAF mutation was detected in both the granuloma and the whole blood of patient #16. ^T599A^B-RAF was detected in the monocytic (CD14+) and lymphoid fractions (CD14−), at a high frequency of 37 to 45% relative mutation abundance, respectively, similar to its abundance in the granuloma (Table 2). This pattern suggested a germ-line mutation. Conventional ‘Sanger’ sequencing of peripheral blood mononuclear cells from the patient and from the patient's mother with an Applied Biosystem Genetic analyzer 3730xl confirmed the presence of an allelic mutation (Figure 1 B). Therefore the ^T599A^B-RAF mutation was present in the peripheral blood of the patient and his mother, indicating a germ-line transmitted allele (Figure 1 C).

Altogether, these data indicate that ^V600E^B-RAF and ^600DLAT^B-RAF are somatic events while ^T599A^B-RAF is a germ-line polymorphism.

### B-RAF 600DLAT is an activating B-RAF mutant

Substitution of Val600 with Glu (^V600E^B-RAF) strongly activates B-RAF [28], [29]. ^600DLAT^B-RAF is an in-frame insertion of 12 nucleotides leading to the insertion of 4 amino acids (Asp-Leu-Ala-Thr, or DLAT) starting from position 600 (Figure 1 A). This insertion, in the structural alignment superimposed to the V600 position, therefore effectively results in a B-RAFV600D substitution, leaving the insertion constituted only by a LATV segment. We have examined the residues surrounding D600 in the structure within a cut-off range of 6 Å. As reported by Wan et al. [29] the V600 residue is in a cluster of hydrophobic residues with Phe468, therefore the presence of a negative charge (residue D) will disrupt this cluster, resulting in destabilization of the inactive conformation of ^600DLAT^B-RAF, exactly as for the V600E mutant (Figure 1 D–F). As expected from our structural modelisation study, ^600DLAT^B-RAF resulted in increased MEK and ERK activation upon transient transfection in 293 T cells in comparison to wild-type B-RAF, similar to what is observed for ^V600E^B-RAF [29] (Figure 1). However, we could not investigate the role of ^V600E^B-RAF and ^600DLAT^B-RAF in myeloid cells, since, when transfected in U937 and THP1 myeloid cell lines both ^V600E^B-RAF and ^600DLAT^B-RAF resulted in growth arrest and cell death (data not shown).

### B-RAF T599A is a dead-kinase B-RAF mutant that does not transactivate C-RAF

**Figure 2:**
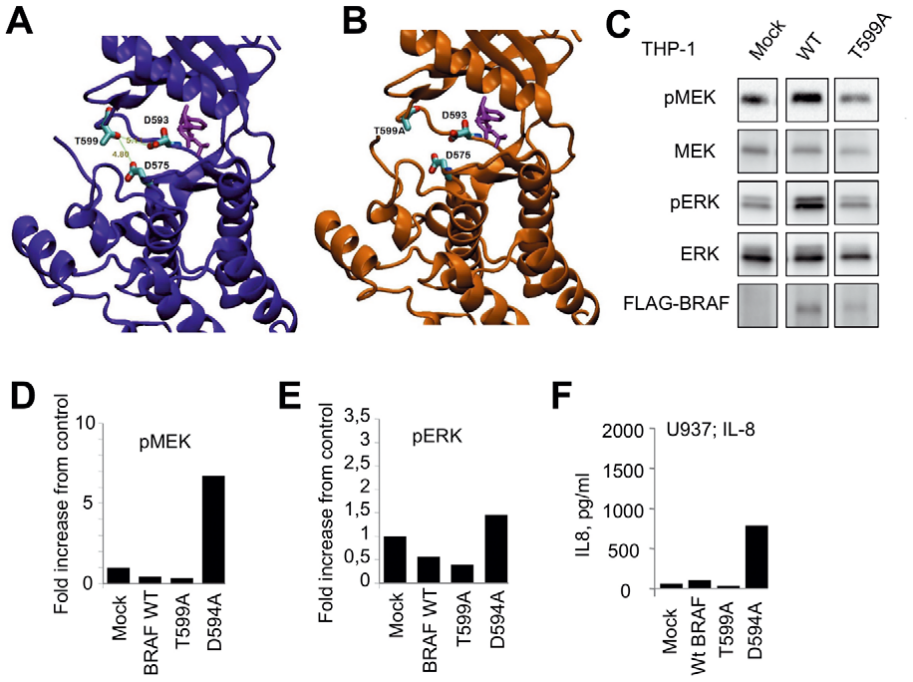
Analysis of ^T599A^B-RAF. (**A, B**) Comparison between models of ^WT^B-RAF 5P_15056 (A, violet) and ^T599A^B-RAF (B, gold). ^T599A^B-RAF substitutes a polar uncharged residue with a hydrophobic residue, causing the loss of short-ranged interactions with residues D576 and D594. **C**. Analysis of MEK and ERK phosphorylation in THP1 cell lines stably transfected with ^WT^B-RAF-FLAG and ^T599A^B-RAF-FLAG. Experiment was repeated twice with similar results. (**D–F**) Analysis of MEK and ERK phosphorylation and IL-8 production in U937 cell lines stably transfected with ^WT^B-RAF, ^T599A^B-RAF, and ^D594A^B-RAF. Experiment was repeated twice with similar results.

Thr599 is a major phosphorylation site in the B-RAF activation domain [28], and substitution of Thr599 with alanine was shown in vitro to suppress B-RAF activity. Indeed, in contrast to ^V600E^B-RAF and ^600DLAT^B-RAF, ^T599A^B-RAF substitutes a polar uncharged residue with a hydrophobic residue, causing the loss of short-ranged interactions with residues D576 and D594 (Figure 1 A, B). This does not predict a destabilization of the inactive conformation of B-RAF. Indeed transfection of ^T599A^B-RAF in 293 T cells did not increase MEK and ERK phosphorylation, in comparison to wt control (Figure 1 G). Co-transfection of C-RAF did not increase MEK and ERK phosphorylation in the presence of ^T599A^B-RAF (Figure 1 H). To further investigate the function of ^T599A^B-RAF in myeloid cells, we retrovirally transfected ^wt^B-RAF and ^T599A^B-RAF in THP1 myeloid cells. As shown in Figure 2 C and 2 D ^T599A^B-RAF did not induce MEK and ERK phosphorylation in THP1 cells above control (Figure 2 C). Thus ^T599A^B-RAF may represent a mutant devoid of intrinsic kinase activity.

However, some B-RAF mutants found in cancer such as ^D594A^B-RAF, albeit devoid of intrinsic B-RAF kinase activity, can transactivate C-RAF and the MEK/ERK pathway [30], [31]. We thus compared the activity of ^T599A^B-RAF with that of ^wt^B-RAF and ^D594A^B-RAF after retroviral transfection into U937 cells. Results indicated that unlike ^D594A^B-RAF, ^T599A^B-RAF did not induce MEK/ERK phosphorylation (Figure 2 D, E) or IL-8 production (Figure 2 F) by U937 cells.

### Clinical features of patients with B-RAF mutations

^V600E^B-RAF mutations were found both in children with granuloma of bones or isolated skin disease, and in infants with early-onset multi-organ disease (Table 1). Among the 16 studied patients, we compared the proportion of patients with or without B-RAF mutations according to the extension of the disease by Fisher exact test and according to the age of diagnosis by Kruskall Wallis Test. No significant difference was observed as the p value was above 0.05 for all tests. However the sample size was small and may not be representative of a population based sample of patients. The patient with a ^600DLAT^B-RAF insertion presented with early-onset multi-organ disease, but responded well to treatment (Patient #10, Table 1). The patient with a germ-line ^T599A^B-RAF allele (Patient #16, Table 1) presented at the age of 10 months with persistent swelling of the left parietal bone. Two months after, clinical examination revealed 10 small skin nodules. Biopsy of one skin element demonstrated the histological diagnosis of LCH with the presence of CD1a+ cells. Patient #16 received therapy by vinblastine and steroid, as per the LCH III protocol, for a total duration of one year. No reactivation of the disease or sequellae was observed during a 7-year follow-up. His mother, who carried the same allelic mutation, is in good health and did not report a personal history of LCH.

## Discussion

In this study we confirm the findings by Badalian-Very et al. [20], that ^V600E^B-RAF mutations are detected in LCH granuloma, and identify two additional mutations ^600DLAT^B-RAF and ^T599A^B-RAF in two LCH patients. The ^600DLAT^B-RAF mutants mimics ^V600E^B-RAF at the structural and functional level. Our results also indicate that ^V600E^B-RAF and ^600DLAT^B-RAF mutations are enriched in CD1a+ ‘LCH’ granuloma cells and absent from the blood of 58 patients, suggesting the presence of somatic mutations in CD1a+ cells, and arguing against a mosaicism in the myeloid lineage or a bone marrow clonal disease, within the sensitivity limits of our deep-sequencing assay. We also identified a novel germ-line ^T599A^B-RAF polymorphism in a patient with LCH, although it remains unclear whether ^T599A^B-RAF is involved in the pathophysiology of LCH in this patient. The data presented here strengthen the association between B-RAF mutation and a dysregulation of the RAS-RAF-MEK pathway in CD1a+ LCH cells from LCH granuloma.

Both the study by Badalian-Very [20] and our present report fail to show a correlation between the presence of B-RAF mutations in LCH granuloma and the patient's age, clinical presentation, or outcome. However, the total number of cases analysed reported (77 in total) is still too small to allow a powerful statistical analysis, given the marked clinical heterogeneity of the disease [1], [4], [5]. A statistical study of the possible association or lack of association of B-RAF somatic mutations with subgroups of LCH patients or with the natural history of the disease remains to be done, in a population based approach.

Our results indicate that both ^600DLAT^B-RAF and ^V600E^B-RAF mutations are somatic events which are not detected in the blood of patients. LCH granulomas may thus arise from a local process within affected tissues, rather than from the continuous recruitment of putative precursors, such as monocytes or dendritic cells carrying activating B-RAFV600E or B-RAF600DLAT alleles. Nevertheless, given the threshold sensitivity of 1%–2% of our deep-sequencing method, we cannot exclude the contribution of a minor myeloid-restricted clone in the bone marrow of patients, or of a subset of patients, and a prospective investigation is required to further investigate whether detection of rare circulating or bone marrow B-RAF mutated cells may be useful to monitor residual disease.

While substitution of Val600 with Glu (^V600E^B-RAF) strongly activates B-RAF, substitution of Thr599 with Ala (^T599A^B-RAF) impairs B-RAF kinase activity [28], [29] given that Thr599 is the major phosphorylation site in the B-RAF activation domain [28]. Other mutations that affect intrinsic B-RAF kinase activity such as ^D594A^B-RAF can also transactivate C-RAF and the MEK/ERK pathway, albeit less strongly [30], [31]. However we have shown here that ^T599A^B-RAF was both impaired in its intrinsic B-RAF kinase activity and unable to transactivate C-RAF. At this time, we do not have a molecular explanation for this observation. Further studies will therefore be needed to potentially unveil the functional consequences of the ^T599A^B-RAF allele on cell activation. The patient's mother born in 1977 carried the same B-RAFT599A allele while in good health, and without a personal history of LCH. Therefore, although the ^T599A^B-RAF allele is not a common polymorphism, there is no evidence that ^T599A^B-RAF is involved in the pathophysiology of LCH in our patient. However the clinical spectrum of LCH is remarkably broad, and the age of onset highly variable [1], [4], [5], preventing us to definitely conclude to the absence of LCH in the mother of the patient. For instance, in one previously reported case of familial LCH, a monostotic lesion was diagnosed in a 20-year-old woman and then also in her daughter when aged 30 months [32].

Finally, ^V600E^B-RAF mutations are observed in a number of benign and tumoral diseases (e.g. naevi and melanoma [25], [26]). In itself this mutation thus do not characterize a malignant disease. It is posssible that the effects of activating B-RAF mutations such as ^V600E^B-RAF and ^600DLAT^B-RAF are different whenever they occur in stem cells or and differentiated cells, and depending on the lineage of the mutated cells, i.e. epithelial or myeloid. Therefore, more work is needed to understand the consequences of ^V600E^B-RAF and ^600DLAT^B-RAF in myeloid cells. We observed that retroviral transduction of either ^V600E^B-RAF or ^600DLAT^B-RAF in U937 and THP1 myeloid cell lines resulted in growth arrest and cell death. This suggests that an in vivo approach may be preferable, since an appropriate cellular environment could be required to support cell growth of ^V600E^B-RAF or ^600DLAT^B-RAF expressing myeloid cells. In this regard, mouse models of conditional expression of B-RAF mutant alleles in different cell lineages [23], should prove extremely useful.

## Materials and Methods

### Patients

Patients were registered in the National French Registry for Langerhans Cell Histiocytosis. LCH diagnosis was established on the basis of the patients' clinical history, histological examination and the mandatory presence of CD1a+ histiocytes in clinical biopsy specimens, and reviewed by a national panel of pathologists. Patient's parents gave written informed consent for the study. The Study was approved by the Institutional Review Board of the University Hospital of Nantes, France. Characteristic of patients 1–16 are summarized in Table 1. Biopsies samples and/or blood were obtained at diagnosis from 15 patients with various clinical forms of the disease, isolated involvement of bone (n=9), early-onset multisystem disease (n=4), isolated skin (n=1) and skin and lung disease (n=1) (Table 1). Purified CD1a+ cells were isolated from biopsies from patients with eosinophilic granuloma of bone (n=3) and early-onset multisystem disease (n=3). Blood was obtain from patient 16 and his parents, and epithelial cells from patient 16, his parents and brother.

### Preparation of DNA from granuloma samples, whole blood, and purified cellular fractions

Samples from CD1a+ LCH cells were obtained as previously described [8]. In brief, after frozen section examination, sterile tissue from eosinophilic granuloma was harvested in RPMI 1640 supplemented with 2 mM L-glutamine, 100 U/mL penicillin, 100 µg/mL streptomycin, and 10% heat-inactivated fetal calf serum (FCS) myoclone (all from GIBCO BRL, Gaithersburg, MD), referred to below as complete medium. Tissues were immediately gently dissociated through a nylon mesh. The cell suspension was washed 3 times and incubated with human IgG to block Fc receptor, and anti-CD1a antibody (BL6; Immunotech, Marseille, France). The cells were washed twice, incubated with antimouse microbeads (MACS; Miltenyi Biotec, Bergisch Gladbach, Germany) for 15 minutes at 4°C. Cells were washed again, and then CD1a+ LCH cells were separated by positive immunomagnetic selection by using a magnetic cell separator (MACS) according to the manufacturer's instructions. Between 2.105 and 6.105 CD1a+ cells were recovered from each sample. Purity of CD1a+ and CD1alow/neg sorted fraction was 80% or greater and mortality 10% or less. Genomic DNA and RNA from frozen granuloma cells, sorted CD1a+ cells and effluent fractions lysed and stored in Trizol were extracted using chloroform, the organic phase was transferred to a fresh tube to extract genomic DNA, and RNA was extracted using the RNA microkit from Qiagen. cDNA was obtained using the Superscript III Reverse-transcriptase kit (Invitrogen) according to the manufacturer's procedure. Genomic DNA extraction from paraffin sections (Patient 16) was performed as per Qiagen protocol for this material. Genomic DNA extraction from whole Blood was performed using the GenElute Blood genomic DNA kit mini prep from Sigma Aldrich according to the manufacturer procedure. Peripheral blood mononuclear cells (PBMCs) were obtained by the standard Ficoll-Hypaque method. CD14+ and CD14− fraction were separated by negative magnetic depletion by using hapten-conjugated CD3, CD7, CD19, CD45RA, CD56, and anti-IgE antibodies (MACS; Miltenyi Biotec) and a MACS according to the manufacturer's instructions. gDNA and RNA were extracted from CD14+ and CD14− MACS purificated monocytes fractions using the RNA/DNA AllPrep QIAgen Minikit (Quiagen).

### B-RAF mutation detection

Pyrosequencing assay was performed using with 454 sequencing (Roche GS FLX platform). Primers sets were designed to amplify B-RAF exons 11 and 15 from both genomic DNA and cDNA, which incorporated a universal forward and reverse sequence tag. A second round of PCR was also performed utilizing the universal tags, to incorporate a sample specific 10 bp “barcode" sequence as well as additional tags utilized in the sequencing process (Roche GS FLX Titanium). Sequencing was performed to yield a ‘depth’ in excess of 500 clonal reads (500×) per exonic amplicon, per patient sample in most cases. This allowed detection of mutant clones down to around 1–2% relative mutation abundance, defined as the proportion of sequence reads that contain the mutation. Independent PCR and GS FLX sequencing experiments were carried out to confirm mutations and to reduce sampling error for calculations of mutation abundance. Somatic mutation in patient #16, his parents and his sibling were confirmed using a Applied Biosystem Genetic analyzer 3730xl (primers available on request).

### B-RAF mutation modeling

The B-RAF patient mutations ^600DLAT^B-RAF and ^T599A^B-RAF have been modeled starting from the structure of the B-RAF kinase domain [29] using the Modeller 9v8 program [33]. The sequence alignment on this domain with the observed patient mutations was performed with the program Praline [34]. The structural alignment of the mutated sequences with the chosen template (pdb code 1WUH) was performed with the program T-coffee [35]. For each mutant, 200 structures have been generated and the ones with the best DOPE score have been selected for further investigations. The VMD program [36] has been used for graphical representations and for structural analyses.

### Transfection of B-RAF alleles in 293 T cells

293 T (Lenti-X 293 T) was purchased from Clontech, and maintained in DMEM supplemented with 10% FBS, 100 units/ml Penicillin, and 100 mg/ml Streptomycin. cDNA fragments of human B-RAF (Genbank accession number: NM_004333) and C-RAF (Genbank accession number: NM_002880) were amplified by RT-PCR from human PBMC cDNA and cloned into pMXs-puro vector. V600E, T599A, D594A, and G596R B-RAF mutants construct were obtained by site-direct mutagenesis (Agilent Technologies). DLAT insertion B-RAF mutant construct was generated by PCR. The constructs were FLAG-tagged and cloned into pMXs-puro and pMXs-IRES-GFP vectors. The mutations were confirmed by DNA sequencing.

293 T was transiently transfected with pMXs-puro, FLAG-B-RAF WT, V600E, T599A, 600DLAT, D564A, G596R, or FLAG-CRAFvectors as indicated in the figure. Transfection was carried out with jetPEI according to the manufacture's instructions (Polyplus transfections, Inc.).

### Generation of stable B-RAF transfectants using retroviral vector

Retrovirus packaging cell line, Plat-A was purchased from Cell Biolabs, Inc., and maintained in DMEM supplemented with 10% FBS, 100 units/ml Penicillin, and 100 mg/ml Streptomycin. U937 was kindly donated by Dr. Taams. THP-1 was kindly donated by Dr. Neil. These cell lines were maintained in RPMI supplemented with 10% FBS, 100 units/ml Penicillin, and 100 mg/ml Streptomycin (complete medium). Plat-A was transiently transfected with pMXs-IRES-GFP, FLAG-B-RAF WT, V600E, T599A, 600DLAT, D594A, and G596R vectors using jetPEI. Twenty-four hours after transfection, the medium was changed to fresh DMEM, and the cells were cultured for further 24 hours. The virus supernatants were collected and the debris was removed by centrifuge. The supernatants were mixed with DOTAP Liposomal Transfection Reagent (Roche) and added to U937 or THP-1 culture, followed by centrifuge at 30°C at 1100×g for 2 hours. Forty-eight hours after infection, GFP positive cells were sorted using BD FACSAria (BD Biosciences) and maintained in complete medium.

### Western-Blot analysis

Twenty-four hours after transfection to 293 T cells, culture medium was changed to serum-free DMEM and cells starved for 18 hours. Cells were lysed with RIPA buffer (20 mM Tris-HCl (pH7.4), 150 mM NaCl, 2 mM EDTA, 1% Nonidet-P 40, 0.1% Sodium dodecyl sulfate, 0.1% Sodium deoxycolate, 50 mM Sodium fluoride, 1 mM b-Glycerophosphate, 1 mM Sodium orthovanadate, 1 mM Phenylmethylsulfonyl fluoride, and Protease inhibitor cocktail (Sigma-Aldrich)). For the experiment using THP-1 transfectant, 1×10^6^ of growing cells were colllected by centrifuge and lysed in RIPA buffer. The cell lysates were separated on 10% SDS-PAGE gel and transferred onto nitrocellulose membranes (Bio-Rad). The membrane were blotted with anti-phospho-MEK1/2 mAb (41G9), anti-MEK1/2 mAb (L38C12), anti-phospho-ERK1/2 (D13.14.4E), anti-ERK1/2 mAb (L34F12) all from Cell Signaling Technology, Inc., and anti-FLAG M2 (Sigma Aldrich). Horseradish peroxidase-coupled secondary antibodies were used to detect the primary antibodies. Signal was revealed by enhanced chemiluminescence (SuperSignal West Pico Chemiluminescent Substrate, Pierce) using a molecular imager Chemidoc™ XRS+, Biorad. Band intensity was quantified by ImageLab™ Analysis Software.

### Quantification of pMEK, pERK, and IL-8 from U937 transfectant by Bio-Plex

For the measurement of IL-8 secretion, 1×104 of U937 transfectants ware laid in a 96 well plate in 100 microL. After 24 hours, the plate were centrifuged and supernatants were collected. For pMEK and pERK, 1×104 of U937 were lysed using Bio-Plex Cell Lysis kit. The samples were analyzed according to manufactures instructions.

### Statistics

Unsupervised students t-test on single comparisons was compared to analyse significance. A P value<0.05 was considered significant.
