# Correction: B-RAF Mutant Alleles Associated with Langerhans Cell Histiocytosis, a Granulomatous Pediatric Disease

**DOI:** 10.1371/annotation/74a67f4e-a536-4b3f-a350-9a4c1e6bebbd

**Published:** 2012-06-28

**Authors:** Takeshi Satoh, Alexander Smith, Aurelien Sarde, Hui-chun Lu, Sophie Mian, Celine Trouillet, Ghulam Mufti, Jean-Francois Emile, Franca Fraternali, Jean Donadieu, Frederic Geissmann

The fifth author's name was misspelled. The correct name is: Syed Mian.

